# Anti-Melanogenic and Antioxidant Activity of *Bifidobacterium longum* Strain ZJ1 Extracts, Isolated from a Chinese Centenarian

**DOI:** 10.3390/ijms241612810

**Published:** 2023-08-15

**Authors:** Jing Wu, Funa Zhang, Haixia Yu, Shimei Qi, Yu Wu, Weihua Xiao

**Affiliations:** 1 The CAS Key Laboratory of Innate Immunity and Chronic Disease, Institute of Immunology, School of Life Sciences, Division of Life Sciences and Medicine, University of Science and Technology of China, Hefei 230027, China; 2 Engineering Technology Research Center of Biotechnology Drugs Anhui, University of Science and Technology of China, Hefei 230027, China; 3 Institute of Advanced Technology, University of Science and Technology of China, Hefei 230094, China

**Keywords:** pigmentation, *Bifidobacterium longum*, anti-melanogenic, tyrosinase, zebrafish, antioxidant

## Abstract

Melanin produced by melanocytes protects our skin against ultraviolet (UV) radiation-induced cell damage and oxidative stress. Melanin overproduction by hyperactivated melanocytes is the direct cause of skin hyperpigmentary disorders, such as freckles and melasma. Exploring natural whitening agents without the concern of toxicity has been highly desired. In this study, we focused on a *Bifidobacterium longum* strain, ZJ1, isolated from a Chinese centenarian, and we evaluated the anti-melanogenic activity of the distinctive extracts of ZJ1. Our results demonstrated that whole lysate (WL) and bacterial lysate (BL) of ZJ1 ferments efficiently reduce α-melanocyte-stimulating hormone (α-MSH)-induced melanin production in B16-F10 cells as well as the melanin content in zebrafish embryos. BL and WL downregulate melanogenesis-related gene expression and indirectly inhibit intracellular tyrosinase activity. Furthermore, they both showed antioxidant activity in a menadione-induced zebrafish embryo model. Our results suggest that ZJ1 fermentation lysates have application potential as therapeutic reagents for hyperpigmentary disorders and whitening agents for cosmetics.

## 1. Introduction

Skin colour is determined mainly by genetic and epigenetic variations as well as UV radiation. Upon UV exposure, skin cells especially keratinocytes from the first layer of the epidermis secrete melanocyte-stimulating hormone (MSH) to initiate melanogenesis in melanocytes, located in the basal layer of the epidermis [1]. Melanocytes and dermal fibroblasts could also produce MSH when stimulated by UV radiation or immune cytokines [2,3]. Melanin pigments are synthesized in specialised organelles within melanocytes, namely, melanosomes, and transferred to keratinocytes [4]. Overproduction and accumulation of melanin or defective melanosome trafficking may induce hyperpigmentary disorders, such as melasma, freckles, age spots and other hyperpigmentation diseases.

Melanogenesis is mainly regulated by several melanogenesis-related proteins including the principal melanogenesis-regulating factor microphthalmia-associated transcription factor (MITF), tyrosinase (TYR), tyrosinase-related protein-1 (TRP-1) and tyrosinase-related protein-2 (TRP-2) [5]. The known whitening agents, e.g., hydroquinone (HQ), 1-phenyl-2-thiourea (PTU) and arbutin, inclusively inhibit tyrosinase activity with nonnegligible side effects, mostly in the form of skin irritation [6,7,8,9]. Thus, natural product-derived agents with high efficacy and satisfactory safety profiles are highly desired for development as therapeutic reagents for hyperpigmentary disorders or as whitening ingredients of cosmetics.

Agents that monitor the transfer of melanin from melanocyte to keratinocyte can also play a role in attenuating hyperpigmentation [10]. For instance, niacinamide [11], as well as its derivative [12], inhibits the transfer of melanosome to keratinocyte. Retinoids alleviates hyperpigmentation by accelerating cell turnover and promoting rapid loss of melanin through epidermopoiesis [13]. Moreover, some specific proteins or enzymes could decolourise melanin in situ, e.g., Laccase from *Trametes versicolor* [14] and Lignin peroxidase [15], offering an alternative strategy to traditional whitening agents.

The gut–skin axis has been extensively investigated in recent years, which highlights that the gut microbiome has both beneficial and adverse impacts on normal physiology and homeostasis of both gut and skin tissues [16,17,18]. *Enterococci, lactobacilli* and *bifidobacteria* are natural inhabitants of the human intestinal tract. They are defined as probiotics [19], bringing health benefits to not only the human gastrointestinal system but also the immune system as well as the skin [20]. The products or fractions from inviable probiotics, namely, postbiotics [19], may exert distinctive health benefits through oral administration [21]. Intriguingly, inanimate fermented products mainly derived from lactic acid bacteria and *S. cerevisiae,* have been developed as cosmetic ingredients [20]. Postbiotics consist of dead microorganisms, fractions, lysates, or metabolisms secreted by live microorganisms or released by cellular lysis. This includes various enzymes, peptides, cell membrane or cytoplasmic proteins, cell wall fractions, polysaccharides and organic acids. There is growing scientific evidence highlighting the skin benefits of postbiotic-derived cosmetics, e.g., antioxidant properties and protection against UV radiation, anti-inflammatory effects, skin microbiota equilibrium and skin barrier/immunity boost, as well as anti-ageing effects [20]. Nevertheless, the anti-melanogenic potential of postbiotics is less well explored.

*Bifidobacterium longum* represents the most abundant species in the neonatal gut, but its abundance declines with age [22,23]. *B. longum* CCFM1029 mediates tryptophan metabolism to improve atopic dermatitis via the gut–skin axis [24]. It potently protects against UV-induced damage in the skin by showing strong anti-immunosuppressive activity and increasing cellular repair. *B. longum* strain ZJ1 was isolated from the intestinal tract of a Chinese centenarian [25]. The potential health benefits of this strain to the gut system and other organs of the human body remain mysterious. Considering the direct association of gut bacteria with skin health as well as the application potential of postbiotics derived from *B. longum* [18,23,26], it is highly interesting to explore the bioactivities of ZJ1 ferments on the skin.

In this study, we first optimised the fermentation parameters of ZJ1 and obtained different extracts of its fermentation product. We then revealed the anti-melanogenic activity of the whole lysate and bacterial lysate on skin melanoma cells and zebrafish embryos with an indirect inhibitory action on tyrosinase. Moreover, the antioxidant properties of ZJ1 lysates were identified in the zebrafish embryo model.

## 2. Results

### 2.1. Preparation of ZJ1 Extracts

We first optimised the manufacturing parameters (i.e., pH, inoculation volume, culture time) of ZJ1 (Figure S1). With the established protocol (initial pH: 6.0 ± 0.4, inoculation volume: 2–4%, culture time: 18–24 h), we obtained four extracts with stable and high yields (Table 1). The cell-free supernatant (CFS, 11.82 ± 0.94 mg/mL) and bacteria were separated through centrifugation. The bacterial pellet was then sonicated to disintegrate the bacteria to obtain soluble cytoplasm components, cell wall fractions and intracellular metabolites, namely bacterial lysate (BL, 9.56 ± 1.12 mg/g). Whole lysate (WL, 11.75 ± 0.93 mg/mL) is a clarified suspension after ultrasonic treatment of the whole fermented product, composed of CFS and BL. Crude polysaccharide (PS, 0.16 ± 0.03 mg/mL) was purified from the cell-free supernatant (see Section 4).

### 2.2. Anti-Melanogenic Effect of ZJ1 Extracts on B16-F10 Cells

We then examined the toxicity of the four extracts on B16-F10 melanoma cells with an MTT assay. BL and PS did not induce observed toxicity on B16-F10 cells when their concentrations did not exceed 200 µg/mL, while WL and CFS treatment showed no apparent cytotoxicity at concentrations of 625 µg/mL (Figure S2).

**Figure 1:**
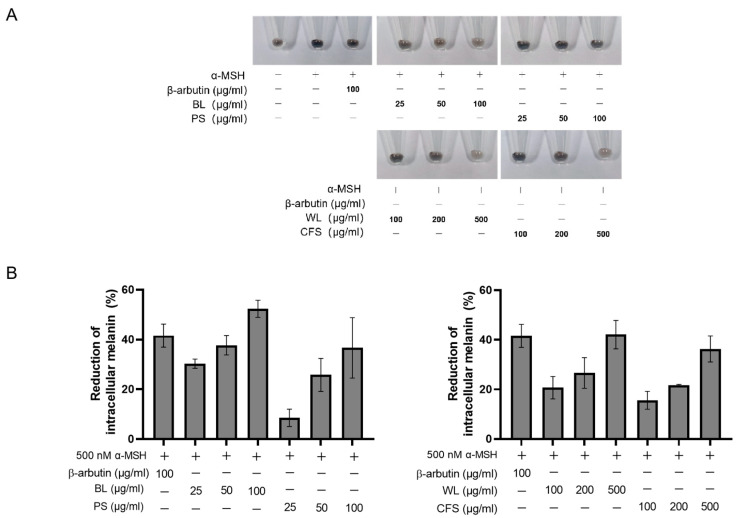
ZJ1 extracts decreased intracellular melanin in α-MSH-stimulated B16-F10 cells. B16-F10 cells were stimulated with α-MSH in the presence of extracts at the indicated concentration for 96 h. (**A**) Cell pellets of B16-F10 cells. (**B**) Intracellular melanin was quantified, and the histograms represent the mean ± s.e.m. of each extract from at least 3 batches.

Subsequently, the anti-melanogenic activity of four extracts was examined in B16-F10 cells at doses without toxicity to the cells (Figure 1). Melanin of B16-F10 cells was stimulated by α-MSH in the presence of ZJ1 extracts, and intracellular melanin content was measured using the melanin content assay. The results demonstrated that the four extracts all reduced intracellular melanin content in a dose-dependent manner. PS and CFS exhibited moderate inhibitory activity, while BL (52.4 ± 3.4% at 100 μg/mL) and WL (42.1 ± 5.7% at 500 μg/mL) reduced cellular melanin to the extent of the positive control, β-arbutin (41.6 ± 4.6% at 100 μg/mL) (Figure 1). β-arbutin can inhibit tyrosinase activity and interfere with the uptake of tyrosine into melanocytes [7]. Moreover, at their concentrations efficiently reducing intracellular melanin content in B16-F10 cells, BL and WL did not have cytotoxicity on human skin keratinocyte HACAT cells (Figure S3) and human fibroblast cells WI-38. These data suggest the anti-pigmentation potential of BL and WL with low toxicity on the skin.

**Figure 2:**
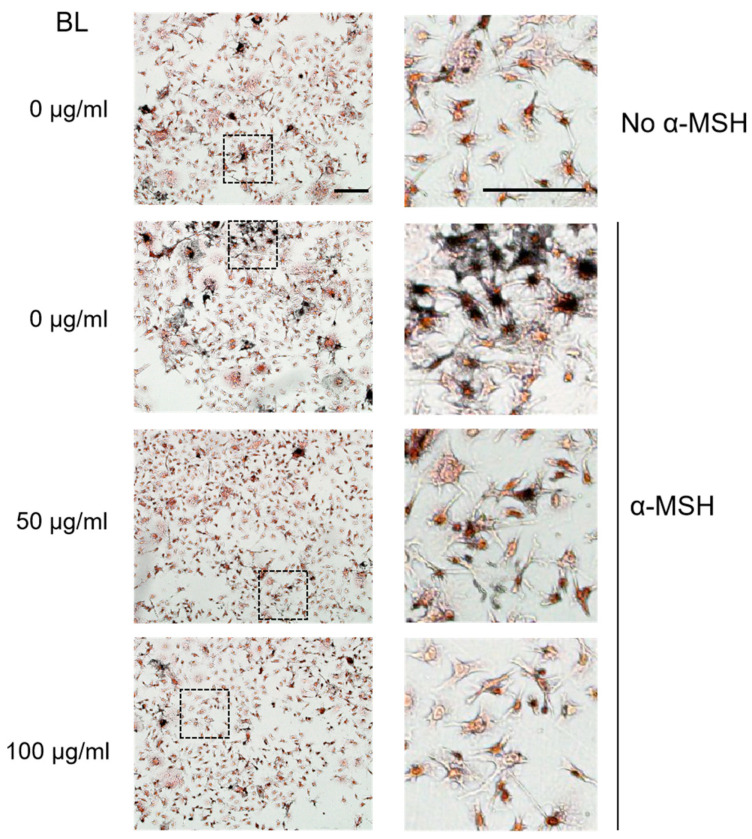
Bacterial lysate (BL) from ZJ1 decreased intracellular melanin in α-MSH- stimulated B16-F10 cells. Representative images show the morphology and intracellular melanin granules of B16-F10 cells stained with Masson–Fontana under the indicated conditions. The right panels are the zoom of the dashed-lined boxes from the left images, scale bar, 100 μm.

Consistent with the quantitative results of melanin reduction in the presence of ZJ1 extracts, microscopic observation of intracellular melanin stained with Masson–Fontana confirmed that BL (100 μg/mL) substantially decreased α-MSH-induced melanin upregulation (Figure 2).

### 2.3. BL and WL Reduce Intracellular Tyrosinase Activity

**Figure 3:**
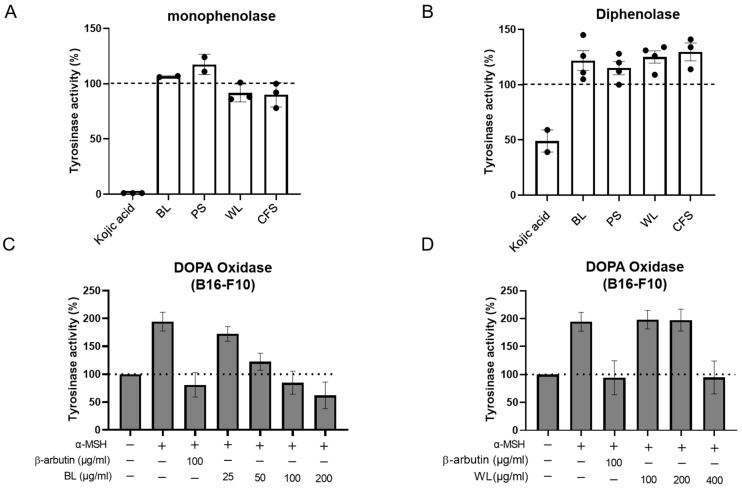
ZJ1 extracts inhibited intracellular tyrosinase activity. Monophenolase (**A**) and diphenolase (**B**) activities were determined using the substrates L-tyrosine and L-DOPA, respectively, and mushroom tyrosinase in the presence of ZJ1 extracts (BL, PS: 125 μg/mL; WL and CFS: 625 μg/mL). Kojic acid (25 μg/mL) was used as a positive control. (**C**,**D**) Intracellular tyrosinase activity of B16-F10 cells treated with BL (**C**) or WL (**D**) in the presence of α-MSH as shown in Figure 1. L-DOPA was used as substrate to determine DOPA oxidase activity of intracellular tyrosinase. Data are represented as the mean ± s.e.m. of 3–4 independent experiments.

The melanogenesis process is mainly catalysed by tyrosinase. Tyrosinase catalyses the conversion of tyrosine to L-DOPA which finally converts to DOPA quinone. Thus, we questioned whether the reduction in cellular melanin by BL and WL is due to the inhibition of tyrosinase activity. To address this issue, we first measured the oxidation of L-tyrosine and L-DOPA by mushroom tyrosinase in the presence of extracts. The data showed that BL and WL at concentrations that significantly reduced cellular melanin in B16-F10 cells did not inhibit tyrosinase (i.e., monophenolase and diphenolase), while kojic acid (25 μg/mL) inhibited 100% of the activity of monophenolase and 51% of diphenolase (Figure 3A,B).

Subsequently, cell lysates of the B16-F10 cells treated with α-MSH and ZJ1 extracts, as performed in Figure 1, were employed as the tyrosinase source to examine the effect of ZJ1 extracts on intracellular tyrosinase activity, instead of mushroom tyrosinase. Coincident with the anti-melanogenic activity in B16-F10 cells, BL (25~200 μg/mL) and WL (100~400 μg/mL) both inhibited α-MSH-induced cellular tyrosinase activity upregulation in a dose-dependent manner, and the maximal inhibitory extents were comparable to that of β-arbutin (Figure 3C,D). Additionally, we observed a similar inhibitory potency of BL and WL on cellular tyrosinase activity from the cells without stimulation of α-MSH (Figure S4). These data suggest that BL and WL both exert indirect actions to inhibit cellular tyrosinase activity rather than inhibiting tyrosinase directly.

### 2.4. BL and WL Downregulate Melanogenesis-Related Gene Expression

**Figure 4:**
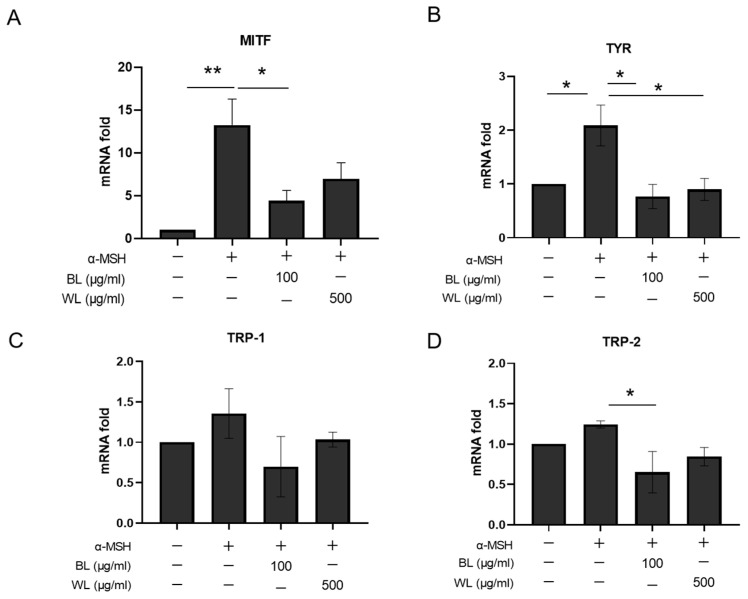
BL and WL monitor melanogenesis-related gene expression in B16-F10 cells. B16-F10 cells were exposed to 500 nM α-MSH in the presence of BL (100 μg/mL) or WL (500 μg/mL), and then mRNA levels were determined with real-time RT-PCR. (**A**) *Mitf*, 12 h after treatment with BL and FL. (**B**–**D**) *Tyr*, *Tyrp1* and *Tyrp2*, 72 h after treatment with BL and FL. The histograms represent the mean ± s.e.m. of 3 independent experiments. *, *p* < 0.05, ** *p* < 0.01, one-way ANOVA.

To further define the anti-melanogenic mechanism of ZJ1 extracts, notably BL and WL, we compared the mRNA levels of four melanogenesis-related genes (i.e., *Mitf, Tyr, Trp-1* and *Trp-2*) in B16-F10 cells after treatment with BL and WL. Figure 4 shows that α-MSH significantly increased the mRNA expression of *Mitf* and *Tyr* as expected, which was substantially reversed by incubation with BL and WL (Figure 4A,B). Moreover, BL was more efficacious in monitoring the levels of *Trp-1* and *Trp-2* (Figure 4C,D). These data suggest that BL and WL may modulate the expression of melanogenesis-related genes in α-MSH-stimulated B16-F10 cells thereby inhibiting tyrosinase activity and melanin production.

### 2.5. BL and WL Inhibit Melanogenesis in Zebrafish Embryos

**Figure 5:**
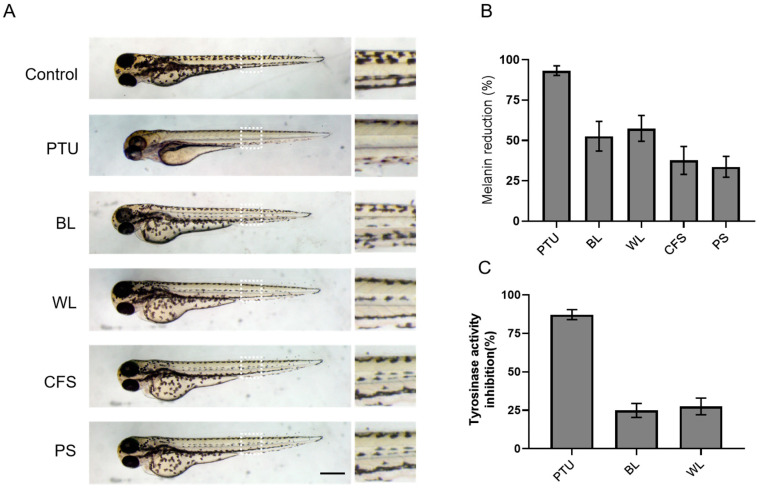
The inhibitory effects of the ZJ1 extract on melanogenesis in zebrafish larvae. Zebrafish larvae (24 hpf) were incubated with extracts at 80 μg/mL for 48 h, and PTU (30 μg/mL) was used as the positive control. (**A**) Representative bright-field images showing the pigmentation of zebrafish embryos treated with ZJ1 extracts at 72 hpf. Right panels are the zoom of the white dashed line. Scale bar, 100 μm. (**B**) The melanin content reduction (%) and (**C**) the inhibition of tyrosinase activity (%) of ZJ1 extracts on zebrafish larvae were quantified and normalised to the non-treated control. The histograms represent the mean ± s.e.m. of 3~4 independent experiments.

To further explore the anti-melanogenic potency of ZJ1 in vivo, we next investigated its antipigmentation activities in the zebrafish embryo model. Zebrafish larvae offer a biologically relevant model for screening antipigmentation agents because of the convenience of visualizing pigment cells through transparent larvae skin and the conserved roles of zebrafish melanophore genes corresponding to mammalian melanocyte genes [27]. Embryos at 24 hpf (hours post-fertilisation) were incubated with BL or WL at 80 μg/mL, a nontoxicity dose in 6-well plates. The melanin content was examined after 48 h. The imaging results showed that, at 72 hpf, the pigmentation was pronounced in the nontreated control group, while the body pigmentation was significantly decreased by PTU. PTU is a tyrosinase inhibitor [6], extensively used to block pigmentation in zebrafish embryos. Importantly, ZJ1 extracts, especially BL and WL, moderately attenuated the pigmentation of zebrafish (Figure 5A). The quantification of total melanin from zebrafish embryos showed that BL reduced 52.6 ± 9.2% of melanin and WL reduced 57.5 ± 8.0% of melanin content, more efficacious than the other two extracts, CFS (37.6 ± 8.7%) and PS (33.7 ± 6.4%) (Figure 5B). Moreover, the intracellular tyrosinase activity from the lysates of BL-treated or WL-treated zebrafish was reduced by 24.9 ± 4.6% and by 27.5 ± 5.4%, respectively (Figure 5C). The data demonstrated the favourable anti-melanogenic efficacy of BL and WL in vivo.

### 2.6. Antioxidant Property of ZJ1 Extracts

Melanogenesis can be induced by sun exposure, medications, hormones, immune cytokine release, etc. [3]. Melanin synthesis is a tyrosinase-catalysed oxidative process with L-tyrosine and L-DOPA as the precursor. Thus, the anti-melanogenic activity of the agents is probably related to their antioxidant activity. The antioxidant effect of ZJ1 extracts was first investigated in vitro using an ABTS (2,2′-azino-bis (3-ethylbenzothiazoline-6-sulphonic acid) radical-scavenging activity assay. BL and WL reduced the ABTS radical level in a dose-dependent manner (Figure S5) and the doses to scavenge ABTS radicals (100–1000 μg/mL) correspond to doses that reduce melanin content in cells. Next, we explored whether BL and WL could inhibit oxidative stress in a zebrafish model at doses that BL and WL efficiently inhibit pigmentation in vivo.

**Figure 6:**
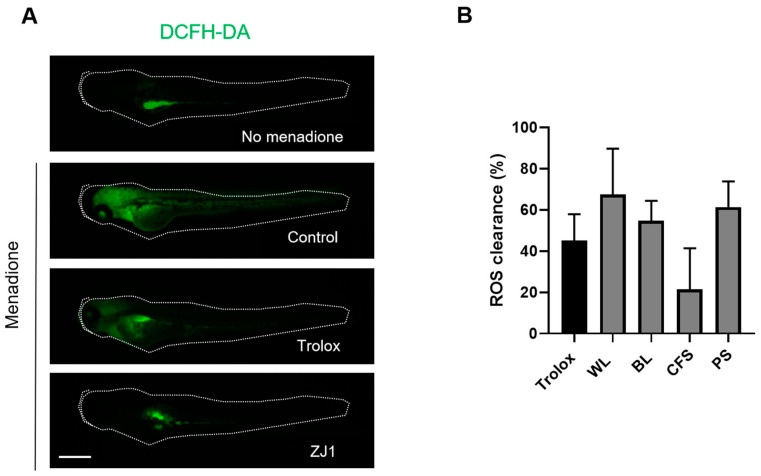
Antioxidant activity of ZJ1 extracts in a zebrafish embryo model. (**A**) Representative images of DCFH-DA-loaded zebrafish embryos treated with positive control Trolox (10 μg/mL) or ZJ1 extracts (25 μg/mL) in the presence of menadione to induce oxidative stress. No menadione addition was considered as the basal condition. Scale bar, 100 μm. (**B**) The histograms represent the mean ± s.d. of ROS clearance (%) from 8 replicates of one representative experiment (*n* = 3).

Reactive oxygen species (ROS) are indispensable in the regulation of the immune system and are one of the key inflammatory mediators. They are also highly associated with melanogenesis. The antioxidant activity of the extracts was investigated in zebrafish embryos in which menadione was employed to induce oxidative stress, and ROS levels were measured using dichloro-dihydro-fluorescein diacetate (DCFH-DA) as a probe. Compared with the basal condition, menadione substantially increased the fluorescence intensity of DCFH-DA (Figure 6A), indicating a significant upregulation of ROS. Notably, the excessive ROS production by menadione following the ZJ1 treatment was substantially reduced, as same as Trolox, a cell-permeable, water-soluble derivative of vitamin E with antioxidant property (Figure 6B). The quantitative results showed that WL (67.6 ± 22.1%) and BL (54.7 ± 9.7%) could efficiently prevent menadione-induced ROS accumulation in vivo, which highlighted the antioxidant potency of ZJ1 in vivo.

## 3. Discussion

In this study, we explored the skin health benefits of the centenarian-originated *Bifidobacterium longum* strain ZJ1. Our results demonstrate that bacterial lysate and whole lysate of ZJ1 have favourable anti-melanogenic activities as well as antioxidant activities in vitro and in a zebrafish embryo model. Importantly, they both inhibit intracellular tyrosinase activity and decrease α-MSH-induced upregulation of melanogenesis-related protein transcription (i.e., MITF, TRY) in B16-F10 cells, suggesting that they may exert an indirect action to attenuate tyrosinase activity rather than inhibiting tyrosinase activity directly. Notably, melanogenesis occurs in melanocytes, which are found in the basal layer of the skin epidermis. Thus, the absorption of ZJ1 extracts, across the stratum corneum and into viable epidermal melanocytes, requires further study in a 3D human skin equivalent [28].

Increasing evidence has revealed that bacterial compounds such as cell wall constituents, products of metabolites, cytoplasm fractions and dead bacteria can elicit certain immune responses in the skin and improve skin barrier functions [16,18,19,20]. Cell-free culture supernatants of multiple probiotics have been extensively studied and they exert significant health benefits on the skin. The cell-free culture supernatant (CFS) of *B. infantis* strain YLGB-14969, isolated from human breast milk, possesses antioxidant and skin barrier-enhancing efficacy in vitro [29]. In particular, the CFS of *B. adolescentis* (BCRC 14658) [30] and *B. bifidum* strain (BCRC 11844) [31] has the capacity to inhibit melanogenesis in melanoma cells with undefined mechanisms.

In our study, we compared the anti-melanogenic and antioxidant activities of four fermentation products, and the results showed that WL and BL both have favourable anti-melanogenic activity and antioxidant activity. The BL is mainly composed of cell wall constituents, cytoplasm fractions and intracellular polysaccharides and metabolisms; thus, there are complexities in defining the active components from the lysate. Lipoteichoic acid (LTA) is a major component of the cell wall of gram-positive bacteria [32]. Its effects on living organisms are different from those of lipopolysaccharide (LPS) found in gram-negative bacteria [33]. LTA contributes to immune regulatory effects as well as anti-ageing effects. LTA isolated from *Lactobacillus plantarum* (pLTA) inhibited melanogenesis in B16-F10 cells by reducing cellular tyrosinase activity and the expression of tyrosinase family members in a dose-dependent manner [33], which is similar to the action of BL from ZJ1.

WL is composed of BL and CFS, containing intracellular metabolites as well as the metabolites released into the medium. Phenyllactic acid, a common metabolite from the CFS of *B. bifidum* and *L. plantarum* by comparative metabolomics analyses, was identified as a tyrosinase inhibitor [34]. It is also worth identifying whether there are specific proteins from ZJ1 fermentation with the enzymatic activity to decolourise melanin [14]. We anticipate that future work will clarify the active components of ZJ1 fermentation products with distinctive activities, which would better define the mechanism of action of ZJ1 extracts and accelerate their development and application. To be noted, Repair Complex CLR™ PF (Chemisches Laboratorium Dr. Kurt Richter GmbH company), a widely used cosmetic ingredient, is a lysate of *B. longum* without defining its active components.

Melanogenesis is a complex biosynthetic process that produces the pigment melanin in human skin. Three melanocyte-specific enzymes, tyrosinase, tyrosinase-related protein 1 (TRP-1) and 2 (TRP-2) are involved in melanogenesis, in which tyrosinase is the key enzyme in the melanin synthesis pathway. Multiple polyphenols and flavonoids, isolated from natural plants or fungi, e.g., arbutin, resveratrol and kojic acid, are representative of tyrosinase inhibitors [35]. Our data suggest that ZJ1 lysate as well as its bacterial lysate, different from known tyrosinase inhibitors, may influence multiple cellular pathways associated with melanogenesis, encompassing the reduction in melanogenesis-related gene transcription and oxidative stress. It is always desirable that diverse active ingredients are combined to achieve a highly synergistic increased efficacy in skin lightening. Nevertheless, the additional potential actions of ZJ1 on melanosome transport inside melanocytes and melanin transfer to keratinocytes as well as the degradation and polarisation of melanin within keratinocytes require further study.

Our data demonstrate that the fermentation extracts of *B. longum* ZJ1 may be potentially applied as a cosmetic ingredient or therapeutic reagent for hyperpigmentary disorders, and additional safety and clinical efficacy tests are needed.

## 4. Materials and Methods

### 4.1. Materials and Reagents

α-MSH (RP10644-5) was purchased from GenScript Biotech (Nanjing, China). ABTS (A109612), tricaine methanesulfonate (E107465), dichloro-dihydro-fluorescein diacetate (DCFH-DA, D103583), β-arbutin (A106856) and Trolox (I2130125) were purchased from Aladdin (Shanghai, China). MTT (M2128), 1-phenyl 2-thiourea (PTU, P7629), L-tyrosine (T8566) and tyrosinase (T3824) were purchased from Sigma-Aldrich (St. Louis, MO, USA). CCK8 assay kit (C0039) was purchased from Beyotime (Shanghai, China). L-DOPA (D807434) and kojic acid (K812221) were from Macklin (Shanghai, China). L-cysteine (CB0132) was purchased from BBI Life Science Products (Shanghai, China), and Man Rogosa Sharp (MRS) Broth (HB0384-1) was from Hopebio (Qingdao, China). Dulbecco’s modified Eagle’s medium (DMEM, C3113-0500) and certified fetal bovine serum (FBS, C04001-500) were purchased from Vivacell (Shanghai, China). Phosphate buffered saline (PBS, BL302A) was from Biosharp (Hefei, China). DMSO (1084ML500) was purchased from BioFROXX (Einhausen, Germany). Penicillin-Streptomycin Solution (100×, P1400) was purchased from Solarbio (Shanghai, China).

### 4.2. Preparation of Bacterial Lysates, Whole Lysates, Cell-Free Supernatant and Polysaccharides

*Bifidobacterium longum* ZJ1 (DDBJ/EMBL/GenBank accession number CP040235) was gifted from Prof. Baolin Sun (USTC, Hefei, China) [25]. The bacterial strain was cultivated in Man Rogosa Sharp (MRS) broth supplemented with 0.5 g/L L-cysteine anaerobically overnight at 37 °C with an optimised protocol.

The cell-free supernatant (CFS) was obtained by centrifugation of a cultured medium at 10,000× *g* for 25 min at 4 °C, and bacteria pellets were washed in PBS and subsequently, ultrasound inactivated followed by centrifugation to obtain supernatant (bacteria lysate, BL). Whole lysate (WL) was collected by centrifugation of an ultrasound-inactivated suspension in the medium at 10,000× *g* for 25 min at 4 °C. All the supernatants were filtered with a 0.22 µm pore size filter and stored at −20 °C until use.

The crude exocellular polysaccharides were purified according to previous approaches with modifications [36]. Briefly, the culture supernatant was first incubated with trichloroacetic acid to precipitate protein, and the polysaccharides were then precipitated from the supernatant with three volumes of absolute cold ethanol for 16 h at 4 °C.

### 4.3. Cell Culture

HaCaT cells (BNCC339817, Bena Culture Collection, Shanghai, China), a spontaneously transformed nontumorigenic human keratinocyte cell line, B16-F10 (CRL-6475, ATCC), were grown in DMEM supplemented with 10% heat-inactivated FBS and antibiotics (100 U/mL penicillin, 10 mg/mL streptomycin). The cells were maintained in tissue culture flasks in a humidified atmosphere of 5% CO_2_ at 37 °C.

### 4.4. Cell Viability Assay

The cytotoxicity of ZJ1 extracts on B16-F10 cells or HACAT was determined with a colorimetric 3-(4,5-dimethylthiazol-2-yl)-2,5-diphenyltetrazolium bromide (MTT) assay or CCK8 assay kit. Cells (1 × 10^4^ cells/well) were seeded on 96-well tissue culture plates overnight. The different extracts of ZJ1 at the indicated concentration in complete medium were added for 48 h. Cells were washed with DPBS once and incubated with complete medium including MTT (final concentration 0.8 mg/mL) for an additional 4 h. Afterwards, 100 μL DMSO was added to dissolve the intracellular insoluble derivative of MTT for 10 min on a shaker, and then the absorbance was measured using an ELISA reader at 490 nm. Cell viability was calculated from the absorbance values relative to control groups and expressed in %.

### 4.5. Assay of Melanin Contents in Melanoma Cells B16-F10

B16-F10 cells (4 × 10^4^ cells/well) were seeded in a 6-well plate and incubated overnight to adhere. The medium of each well was replaced with fresh medium containing ZJ1 extracts as well as 500 nM α-MSH and then incubated for 96 h (change medium with same compounds at 48 h). Then the cells were all washed with PBS and harvested using trypsin to dissociate cells. After centrifugation at 5000 rpm for 5 min, the cell pellets were lysed with 1 N NaOH-containing 10% DMSO in a water bath at 90 °C for 1 h to solubilize melanin. Then 100 μL aliquots were transferred to a 96-well plate and the melanin content was measured according to the absorbance at 405 nm with a SpectraMax iD5 reader (Molecular Devices, San Jose, CA, USA). Cells without treatment of α-MSH were considered as control.

### 4.6. Assay of Cell-Free Mushroom Tyrosinase Activity

Tyrosinase (200 units/mL) 50 μL was mixed with 50 μL ZJ1 extracts or kojic acid (100 µg/mL) as a reference for 10 min in a 37 °C incubator. The reactions were then initiated by adding 100 μL L-tyrosine (0.5 mg/mL) for 60 min or L-DOPA (1 mg/mL) for 30 min. Tyrosinase activity was determined from the absorbance of the mixture at 475 nm, which was measured with a SpectraMax iD5 reader.

### 4.7. Assay of Intracellular Tyrosinase Activity

B16-F10 cells were treated as shown in the Section 4.5 *Assay of melanin contents in melanoma cells B16-F10.* After 72 h, cells were then washed twice with cold DPBS and lysed with phosphate buffer (pH 6.8) containing 0.5% Triton X-100. Cell lysates were clarified by centrifugation at 12,000× *g* for 10 min. Each lysate (100 μL) was placed in a 96-well plate, and a 10 μL aliquot of 2 mg/mL L-DOPA was added to each well. After incubation at 37 °C for 60 min, the absorbance was measured at 405 nm using a SpectraMax iD5 reader. The tyrosinase activity was calculated and normalised to the total protein content, expressed as % of control. The control was cells without incubation with α-MSH and ZJ1.

### 4.8. Reverse Transcriptase-Polymerase Chain Reaction (RT-PCR) and Quantitative Real-Time Polymerase Chain Reaction

Total RNA was isolated from B16-F10 cells using the RNASimple Total RNA Kit (TIANGEN, Beijing, China) according to the manufacturer’s instructions and quantified by measuring the absorbance at 260 nm. RNA was converted to cDNA using a StarScript II RT MasterMix Kit (Genstar, Beijing, China). Specific primers used for RT-PCR were designed according to the method reported by Lee et al. [37] (listed in Table S1). To quantify mRNA expression, real-time PCR amplification was carried out on a CFX96 Real-Time System (Bio-Rad, Hercules, CA, USA) with 2× SYBR Green qPCR Master Mix (Bimake, Houston, TX, USA). All gene expression levels were calculated with the Ct values by the method 2^−∆∆Ct^ (where ∆Ct = Ct [target gene] − Ct [GAPDH]). The expression levels of the tested genes were calculated using the delta Ct method and normalised to the expression levels of GAPDH. Each experiment was performed in triplicate at least twice.

### 4.9. ABTS Assay

ABTS (2,2′-azino-bis(3-ethylbenzothiazoline-6 sulfonic acid) scavenging activity was performed as previously described [38]. Trolox was used as a positive control, and distilled water was used as a blank control. The percentage of ABTS radical scavenging activity with all samples at the indicated concentrations was calculated using the formula.

ABTS radical scavenging activity (%) *=* [1 − (Absorbance of sample)/(Absorbance of control)] × 100%.

### 4.10. Zebrafish Maintenance and Embryo Collection

Adult zebrafish were cultured with a 14 h light/10 h dark cycle in a circulating water system and were fed three times per day. Female and male zebrafish were placed in the breeding tank at a ratio of 2:3, and embryos were collected the next morning and cultured in embryo culture medium at 28.5 °C. Protocols were approved by the Committee on the ethics of Animal Experiments of the USTC (Permit No. USTCACUC1103013).

### 4.11. Assay of Melanin Content and Tyrosinase Activity in Zebrafish Embryos

Briefly, collected zebrafish embryos (24 hpf) were arrayed into a 6-well plate at 15 embryos per well with embryo medium. The prepared ZJ1 extracts were added to the embryo medium and incubated for 48 h. Zebrafish embryos were anaesthetised with tricaine methanesulfonate (150 mg/L) solution and imaged under a SZX7 Stereo-microscope (Olympus, Shinjuku, Japan). ImageJ 1.53c software was used to measure pixels. To measure melanin content, embryos were sonicated in 5 mg/mL sodium deoxycholate and centrifuged to collect the pellets, which were then boiled with 1 N NaOH for 1 h. The absorbance was measured at 405 nm. For the tyrosinase activity assay, 100 µL of supernatant after sonication and centrifugation was mixed with 100 µL of 5 mM L-DOPA and incubated at 37 °C for 1 h. The absorbance at 475 nm was measured.

To be noted, toxicity tests on zebrafish embryos showed that both extracts BL and WL stopped the embryogenesis process when the concentration exceeded 100 μg/mL.

### 4.12. Assay of Cellular Reactive Oxygen Species (ROS) in Zebrafish Embryo Model

The intracellular ROS in zebrafish embryos was detected with an oxidation-sensitive fluorescent probe dye, DCFH-DA. Briefly, embryos at 48 hpf were placed into 6-well plates and incubated with 3 µM menadione in the presence of ZJ1 extracts at a final concentration of 25 µg/mL at 28.5 °C for 22 ± 2 h followed by washing with embryo culture medium. DCFH-DA was added to the well at a final concentration of 10 μg/mL, and the zebrafish embryos were cultured for 1 h in the dark. The fluorescence intensity of DCFH-DA of embryos (each embryo in an individual well of a black 96-well plate) was read at 485/535 nm with the reader SpectraMax iD5. Antioxidant Trolox was used at 10 µg/mL as the positive control.

### 4.13. Statistics

All data are presented as the mean ± s.d. or mean ± s.e.m. unless specified in the figure legends. Student’s *t*-test was applied to determine statistical significance between two datasets, and one-way ANOVA was used to perform analyses between more than two datasets. A value of *p* ≤ 0.05 was considered significant (denoted * *p* < 0.05, ** *p* < 0.01, *** *p* < 0.001). GraphPad Prism 8 software was used to construct the graphs and perform statistical analysis.

## 5. Conclusions

Our work explored the application potential of a *Bifidobacterium longum* strain, ZJ1, isolated from a Chinese centenarian. We first optimised the culture parameters of ZJ1 and obtained four extracts from its fermented products with stable and high yields. Their anti-melanogenic activities were examined in the α-MSH-stimulated melanoma cell model and zebrafish embryo model. The results showed that the bacterial lysate (BL) and the whole lysates (WL) harbour favourable anti-melanogenic activity, and their maximal inhibitory extents were comparable to that of β-arbutin, a tyrosinase inhibitor. The activities of BL and WL are related to the downregulation of melanogenesis-related gene expression and indirect inhibition of intracellular tyrosinase activities, rather than direct actions on tyrosinase. Moreover, BL and WL both exhibit antioxidant activity in the zebrafish embryo model. Overall, our data suggest that ZJ1 fermentation extracts can be developed as therapeutic reagents for hyperpigmentary disorders or as whitening agents for cosmetic use. Further studies to identify the active components of ZJ1 extracts are ongoing.

## 6. Limitations of the Study

Here we report the discovery of a potential whitening agent from *Bifidobacterium longum* strain ZJ1. Its extracts exhibit anti-melanogenic activity in melanoma cells and the zebrafish embryo model. However, there are two main limitations in our study. First, additional works on human primary melanocytes and reconstructed 3D pigmented skin equivalents are required to confirm ZJ1 bioactivities. Secondly, the active components from the ZJ1 fermentation extracts would need to be screened and characterised.

## Figures and Tables

**Table 1:** Contents of protein and polysaccharide in the ZJ1 extracts.

Fraction	Bacterial Lysate (BL) (mg/g, *n* = 9)	Cell-Free Supernatant (CFS) (mg/mL, *n* = 5 or 8)	Whole Lysate (WL) (mg/mL, *n* = 7)
Polysaccharide	n.d	0.16 ± 0.03	n.d
Protein	9.56 ± 1.12	11.82 ± 0.94	11.75 ± 0.93

Values are presented as the mean ± s.d. from 5~9 batches, n.d means not detected.

## Data Availability

The data presented in this study are available on request from the corresponding author.

## Supplementary Materials

The supporting information can be downloaded at: https://www.mdpi.com/article/10.3390/ijms241612810/s1.
