# Supplementary material for: Anti-Melanogenic and Antioxidant Activity of Bifidobacterium longum Strain ZJ1 Extracts, Isolated from a Chinese Centenarian
Source: Int J Mol Sci. 2023 Aug 15;24(16):12810. doi: 10.3390/ijms241612810 (PMC10454566; doi:10.3390/ijms241612810)
Supplement: Supplementary file 1 [file ijms-24-12810-s001.zip › Supplementary information.pdf]

Supplementary information

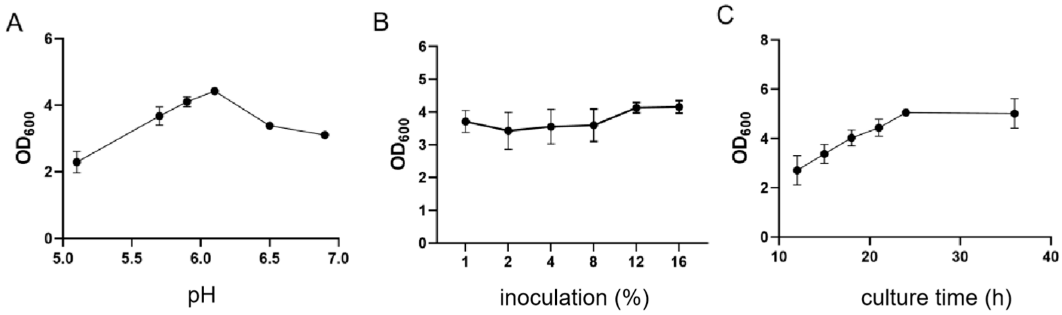

Figure S1. Parameter Optimization of ZJ1 fermentation in MRSL medium. OD_{600} of ZJ1 culture at different pH values (A), inoculation volumes (B) and culture times (C). The results represent the mean ± s.e.m of 3 independent batches (*n* = 3).

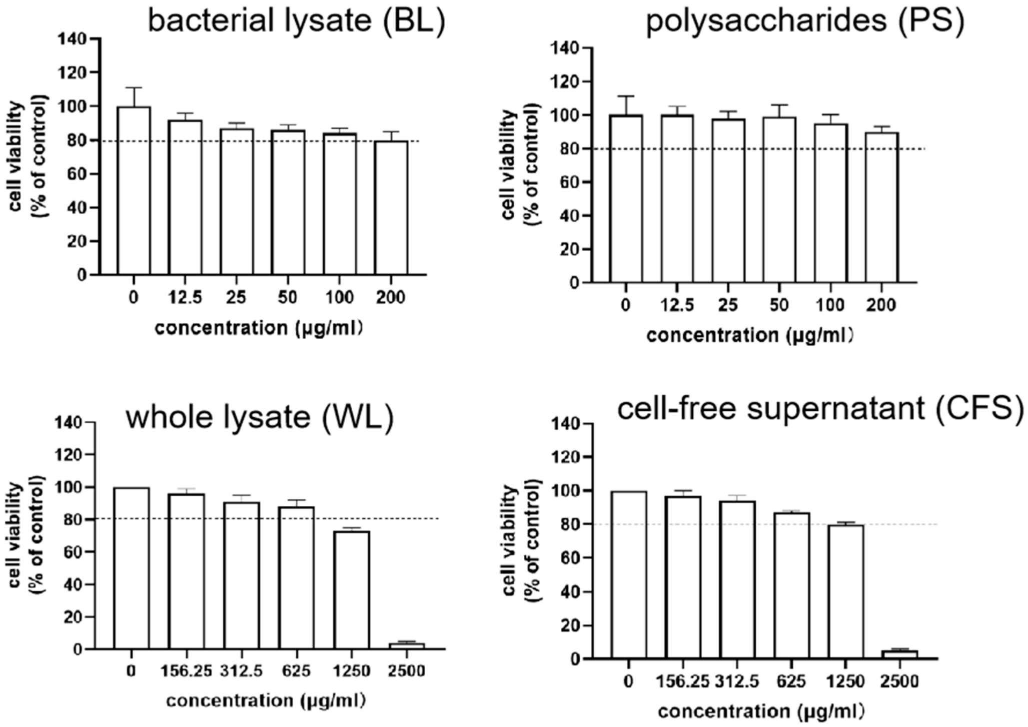

Figure S2. The viability of B16-F10 cells after incubation with different extracts of ZJ1 for 48 h. Viability was determined using the MTT assay, and the histograms represent the mean ± s.d. of quintuplicate wells of one representative experiment (*n* = 3).

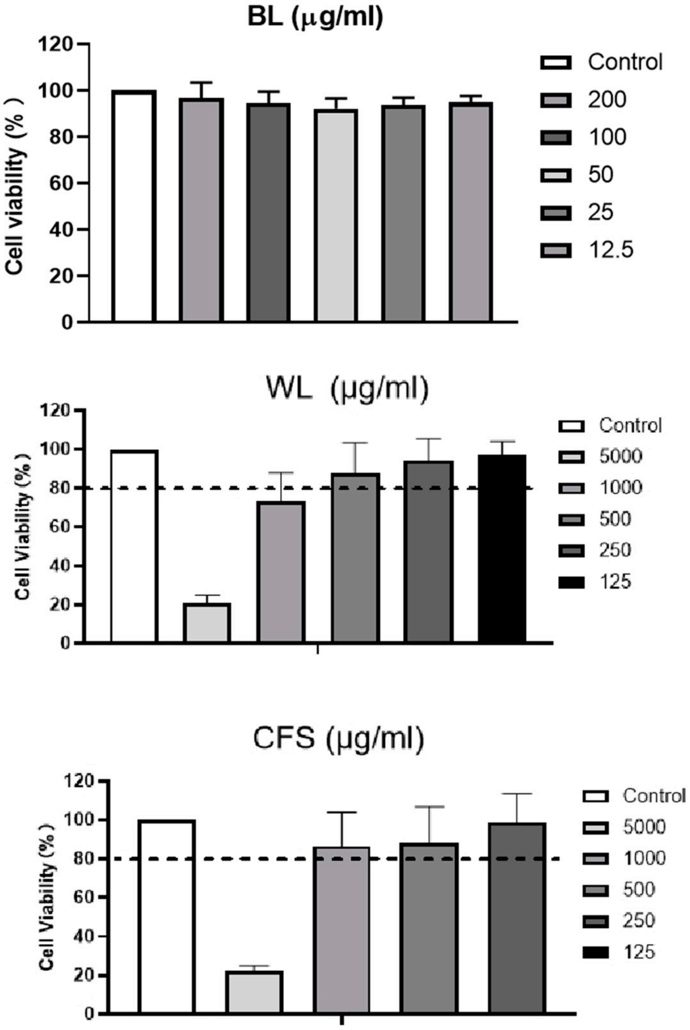

Fig S3. The viability of HACAT cells after incubation with ZJ1 extracts for 48 h. The viability was determined using the MTT assay, and the histograms represent the mean ± s.d. of quintuplicate wells of one representative experiment (*n* = 2).

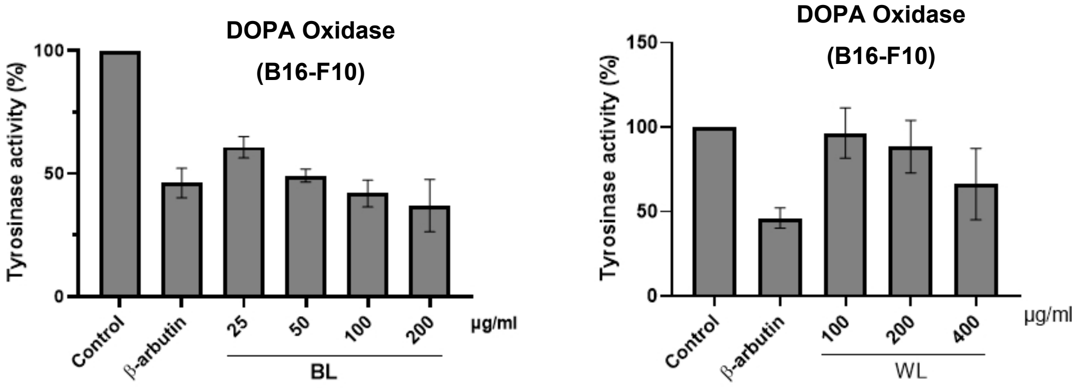

Fig S4. ZJ1 extracts inhibit intracellular tyrosinase activity, corresponding to Fig 3. B16-F10 cells were incubated with BL or WL at the indicated concentrations in the

absence of α-MSH, and then the tyrosinase activity of the cell lysates was measured as shown in Fig 3. Histograms represent the mean ± s.e.m of 3~ 4 replicate experiments.

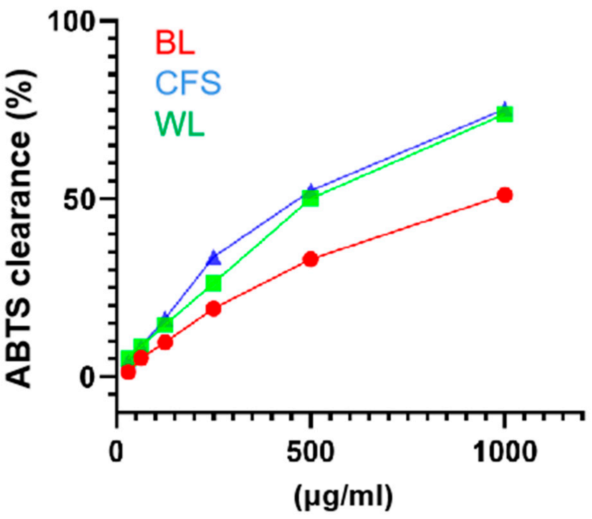

Fig S5. Scavenging activity of ZJ1 extracts at the indicated concentrations on ABTS by free radical scavenging experiments, corresponding to Fig 6.

Table S1. Primer information

Gene	Sequence
GAPDH	Fw: GCCAAACGGGTCATCATCTC
	Rv: GTCATGAGCCCTTCCAVAAT
MITF	Fw: AGGACCTTGAAAACCGACAG
	Rv: GTGGATGGGATAAGGGAAAG
TYR	Fw: AGCCTGTGCCTCCTCTAA
	Rv: AGGAACCTCTGCCTGAAA
TRP-1	Fw: CGATACCCTGGGAACACT
	Rv: TACACGGACCTCCAAGCA
TRP-2	Fw: CCAACGCTGATTAGTCGGA
	Rv: GAAGAAGGGAGGGCTGTCA
